# P063 Beyond giant cell arteritis: recognising herpes zoster ophthalmicus as a vasculitis mimic

**DOI:** 10.1093/rap/rkag113.093

**Published:** 2026-11-03

**Authors:** Nirooshika Ilanko, Elsa Sunny, Alexandra Godlee

**Affiliations:** Queen Elizabeth Hospital Birmingham, Birmingham, United Kingdom; Queen Elizabeth Hospital Birmingham, Birmingham, United Kingdom; Queen Elizabeth Hospital Birmingham, Birmingham, United Kingdom

## Abstract

**Introduction:**

Giant cell arteritis (GCA) is a medical emergency in older adults due to the risk of irreversible visual loss. Prompt initiation of corticosteroids is often recommended when clinical suspicion is high. However, several conditions can mimic GCA, including varicella-zoster virus (VZV) infection, which may present with unilateral headache, raised inflammatory markers, and vascular imaging abnormalities. Distinguishing between these entities is crucial, as management strategies differ significantly. We report a case of herpes zoster ophthalmicus presenting as suspected GCA, highlighting an important diagnostic pitfall in elderly patients.

**Case description:**

A 90-year-old woman presented to the outpatient clinic with a two-week history of right-sided temporal headache associated with localized temporal pain and blurring of vision in the right eye. She denied jaw claudication, diplopia, or scalp dysesthesia. There were no constitutional symptoms such as fever, night sweats, weight loss, or fatigue. She also reported no shoulder or pelvic girdle stiffness suggestive of polymyalgia rheumatica.

On examination, she was afebrile and systemically well. Tenderness was noted over the right temporal artery without nodularity and both temporal artery pulses were palpable. Neurological examination was normal. Musculoskeletal examination was unremarkable. Urgent ophthalmological assessment excluded ischemic optic neuropathy, retinal vasculitis, or other vasculitic ocular involvement.

Laboratory investigations demonstrated mildly elevated inflammatory markers, with an erythrocyte sedimentation rate of 27 mm/1^st^ hour and C-reactive protein of 10 mg/L. Given her advanced age, unilateral temporal headache, raised inflammatory markers, and concern for visual symptoms, a possible diagnosis of GCA was considered. A temporal artery ultrasound revealed focal changes suggestive of arteritis. In view of the potential risk of vision loss, high-dose corticosteroid therapy was commenced.

The patient’s headache initially improved. However, approximately one week after starting corticosteroids, she developed a painful vesicular rash distributed over the right ophthalmic (V1) dermatome. The appearance was clinically consistent with herpes zoster ophthalmicus, and the diagnosis of shingles was confirmed. Corticosteroids were rapidly tapered and discontinued, and antiviral therapy was initiated promptly. The patient did not develop ocular complications related to herpes zoster, and her symptoms gradually resolved.

**Discussion:**

This case was particularly interesting because it demonstrated the diagnostic challenges of distinguishing giant cell arteritis (GCA) from herpes zoster ophthalmicus (HZO), especially in an elderly patient where both conditions can present with unilateral temporal pain and visual symptoms. The initial clinical decision to consider GCA was based on the patient’s advanced age, new-onset temporal headache, localized temporal artery tenderness, visual blurring, mildly elevated inflammatory markers, and temporal artery ultrasound findings suggestive of arteritis.

In suspected GCA, treatment should not be delayed when clinical suspicion is sufficient, even if the presentation is atypical. However, the subsequent development of a painful vesicular rash in the ophthalmic (V1) dermatome highlighted that alternative diagnoses must remain under continuous consideration. The emergence of the rash confirmed herpes zoster ophthalmicus as the underlying diagnosis and prompted rapid reassessment of the management plan.

This case highlights that temporal artery ultrasound, although highly valuable in the diagnosis of giant cell arteritis, is not infallible. False-positive findings may occur because of atherosclerotic vascular changes, technical factors, or other inflammatory conditions. In patients with atypical clinical features and only modest inflammatory marker elevation, ultrasound findings should be interpreted cautiously within the overall clinical context. The subsequent emergence of herpes zoster ophthalmicus in this patient illustrates the importance of serial clinical reassessment and avoiding overreliance on imaging alone when establishing the diagnosis of GCA.

**Key learning points:**

• This case illustrates that herpes zoster, particularly VZV-associated vasculopathy, can closely mimic GCA both clinically and on imaging. The absence of classic GCA features such as jaw claudication, polymyalgia rheumatica, or systemic symptoms should prompt careful reconsideration of the diagnosis. Clinicians should remain vigilant for alternative diagnoses in elderly patients presenting with unilateral headache, as early corticosteroid therapy may unmask or worsen underlying VZV infection. Awareness of this mimic can help avoid unnecessary prolonged immunosuppression and ensure timely antiviral treatment.

• This case has been a valuable learning experience because it highlighted the importance of clinical reasoning in situations where the diagnosis is uncertain and where early treatment decisions carry significant consequences. The main learning point has been that while clinical guidelines appropriately advocate prompt corticosteroid treatment in suspected giant cell arteritis (GCA) to prevent irreversible visual loss, this approach must be accompanied by continuous reassessment as new clinical information emerges. An initial working diagnosis should not become fixed, particularly when the presentation is atypical.

• The importance of considering the pre-test probability of disease rather than relying heavily on a single investigation. Although the temporal artery ultrasound was suggestive of arteritis, the patient had only mildly elevated inflammatory markers and lacked several classical features of GCA, including jaw claudication, constitutional symptoms, and polymyalgia rheumatica. This reinforced the principle that imaging findings should always be interpreted in conjunction with the overall clinical picture.

• Another key learning point was recognizing the value of maintaining a broad differential diagnosis. Herpes zoster ophthalmicus can present with prodromal headache and visual symptoms before the appearance of the characteristic rash, closely mimicking GCA. Remaining alert to evolving clinical signs enabled timely recognition of the correct diagnosis and appropriate modification of treatment.

